# Activation of the hedgehog pathway in advanced prostate cancer

**DOI:** 10.1186/1476-4598-3-29

**Published:** 2004-10-13

**Authors:** Tao Sheng, Chengxin Li, Xiaoli Zhang, Sumin Chi, Nonggao He, Kai Chen, Frank McCormick, Zoran Gatalica, Jingwu Xie

**Affiliations:** 1 Sealy Centers for Cancer Cell Biology and Environmental Health, Department of Pharmacology and Toxicology, University of Texas Medical Branch, Galveston, Texas, 77555-1048, USA; 2 Department of Dermatology, Xijing hospital, Xi'an 710032, China; 3 UCSF Cancer Center, 2340 Sutter Street, San Francisco, CA 94115, USA; 4 Department of Pathology, Creighton University Medical Center, 601 N 30^th ^St. Omaha, NE 68131, USA

## Abstract

**Background:**

The hedgehog pathway plays a critical role in the development of prostate. However, the role of the hedgehog pathway in prostate cancer is not clear. Prostate cancer is the second most prevalent cause of cancer death in American men. Therefore, identification of novel therapeutic targets for prostate cancer has significant clinical implications.

**Results:**

Here we report that activation of the hedgehog pathway occurs frequently in advanced human prostate cancer. We find that high levels of hedgehog target genes, PTCH1 and hedgehog-interacting protein (HIP), are detected in over 70% of prostate tumors with Gleason scores 8–10, but in only 22% of tumors with Gleason scores 3–6. Furthermore, four available metastatic tumors all have high expression of PTCH1 and HIP. To identify the mechanism of the hedgehog signaling activation, we examine expression of Su(Fu) protein, a negative regulator of the hedgehog pathway. We find that Su(Fu) protein is undetectable in 11 of 27 PTCH1 positive tumors, two of them contain somatic loss-of-function mutations of *Su(Fu)*. Furthermore, expression of sonic hedgehog protein is detected in majority of PTCH1 positive tumors (24 out of 27). High levels of hedgehog target genes are also detected in four prostate cancer cell lines (TSU, DU145, LN-Cap and PC3). We demonstrate that inhibition of hedgehog signaling by smoothened antagonist, cyclopamine, suppresses hedgehog signaling, down-regulates cell invasiveness and induces apoptosis. In addition, cancer cells expressing Gli1 under the CMV promoter are resistant to cyclopamine-mediated apoptosis. All these data suggest a significant role of the hedgehog pathway for cellular functions of prostate cancer cells.

**Conclusion:**

Our data indicate that activation of the hedgehog pathway, through loss of Su(Fu) or overexpression of sonic hedgehog, may involve tumor progression and metastases of prostate cancer. Thus, targeted inhibition of hedgehog signaling may have significant implications of prostate cancer therapeutics.

## Background

The *hedgehog *(Hh) pathway plays a critical role in embryonic development and tissue polarity [1]. Secreted Hh molecules bind to the receptor *patched *(PTC-PTCH1, PTCH2), thereby alleviating PTC-mediated suppression of *smoothened *(SMO), a putative seven-transmembrane protein. SMO signaling triggers a cascade of intracellular events, leading to activation of the pathway through GLI-dependent transcription [2]. The hedgehog receptor PTCH1 is also a target gene of this pathway, which forms a negative feedback mechanism to maintain the pathway activity at an appropriate level in a given cell. Activation of Hh signaling through loss-of-function somatic mutations of PTCH1 in human basal cell carcinomas (BCCs) disrupts this feedback regulation, leading to uncontrolled SMO signaling. Activating mutations of SMO in BCCs, on the other hand, are resistant to PTCH1-mediated inhibition, leading to an outcome similar to PTCH1 inactivation [3-6]. More recently, abnormal activation of the sonic hedgehog pathway, through over-expression of sonic hedgehog, has been implicated in the development of subsets of medulloblastomas, small cell lung cancer and gastrointestinal tract (GI) cancers [7-10].

Development of prostate requires hedgehog signaling. Although the initial formation of prostate buds does not require sonic hedgehog signaling (shh), shh is critical for maintaining appropriate prostate growth, proliferation and tissue polarity [11-14]. In the adult prostate, however, the activity of the hedgehog pathway is quite low. It remains to be tested whether this hedgehog pathway is activated during development of prostate cancer, the second most prevalent cause of cancer death in American men. Activation of the hedgehog pathway is often indicated by elevated levels of PTCH1 and HIP. In addition to PTCH1 mutation, SMO activation and hedgehog over-expression, loss of Su(Fu) can result in activation of the hedgehog pathway. In the human, the Su(Fu) gene is localized at chromosome 10q24, a region with LOH in several types of cancer including prostate cancer, lung cancer, breast cancer and medulloblastomas [15,16]. As a negative regulator of the hedgehog pathway, Su(Fu) inhibits the function of Gli molecules, leading to inactivation of this pathway [17-19]. Su(Fu) is also reported to affect beta-catenin function [20]. In addition, over-expression of sonic hedgehog is shown to be involved in the development of GI cancers [9,10]. Here we report our findings that activation of the hedgehog pathway occurs frequently in advanced prostate cancers, possibly through loss of Su(Fu) protein or over-expression of sonic hedgehog.

## Results

### Elevated expression of hedgehog target genes in prostate cancer specimens

As an important regulator of tissue polarity, active hedgehog signaling is required for ductual morphogenesis and proliferation during prostate development [11-14]. The adult prostate, on the other hand, does not contain active hedgehog signaling. Because hedgehog signaling is an important regulator for epithelial-mesenchymal interaction, an event critical during prostate cancer development, we examined whether the hedgehog-signaling pathway is activated in prostate cancer.

**Figure 1:**
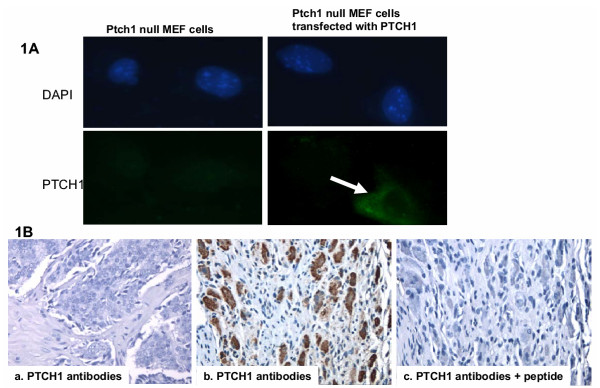
**Detection of PTCH1 expression in prostate cancers. **Protein expression of PTCH1 was detected by immunostaining. PTCH1 antibodies (Santa Cruz Biotechnology Cat# 9149) were tested in *Ptch1*^-/- ^null MEF cells (**A**). While *Ptch1*^-/- ^null MEF cells had no positive fluorescent staining with PTCH1 antibodies, transfection of PTCH1 expressing plasmid lead to positive staining (green, indicated by an arrow, 400×). Immunohistochemistry of prostate cancer specimens with PTCH1 gave negative (**B-a, **200×) or positive (Red in **B-b, **200×) signals. When PTCH1 antibodies were pre-incubated with the very peptide for raising the antibodies, no positive signals could be observed (**B-c**).

Activation of hedgehog signaling causes elevated expression of target genes PTCH1 and HIP. Thus, increased protein expression of PTCH1 and HIP indicates activation of the hedgehog pathway. Using PTCH1 antibodies [10], we examined 59 prostate cancer samples for hedgehog signaling activation (see Table 1, Additional file 1 for details). We first tested the specificity of the PTCH1 antibodies in MEF cells. *Ptch1 *null MEF cells have no active *Ptch1 *gene, thus should not have positive staining with PTCH1 antibodies. Indeed, no staining was seen in *Ptch1 *null MEF cells (Fig. 1A). After transfection of *PTCH1 *expressing plasmid, transfected cells showed positive staining (Fig. 1A), indicating that the PTCH1 antibodies are specific to PTCH1. Furthermore, PTCH1 immunohistostining was abolished after addition of the specific peptide, from which the antibodies were raised (Fig. 1B,1c). We found that percentage of PTCH1 positive staining tumors increased in high grade tumors (Table 1, Additional file 1). In prostate cancers with Gleason scores 3–6, 4 out of 18 specimens were positive for PTCH1 (22%), whereas 16 out of 22 undifferentiated carcinomas (Gleason Scores of 8–10) expressed PTCH1 (73%, see Table 1, Additional file 1), suggesting that the hedgehog pathway is frequently activated in advanced prostate cancer. To confirm this data, we found that all four available metastatic prostate cancer specimens were all positive for PTCH1 staining.

**Figure 3:**
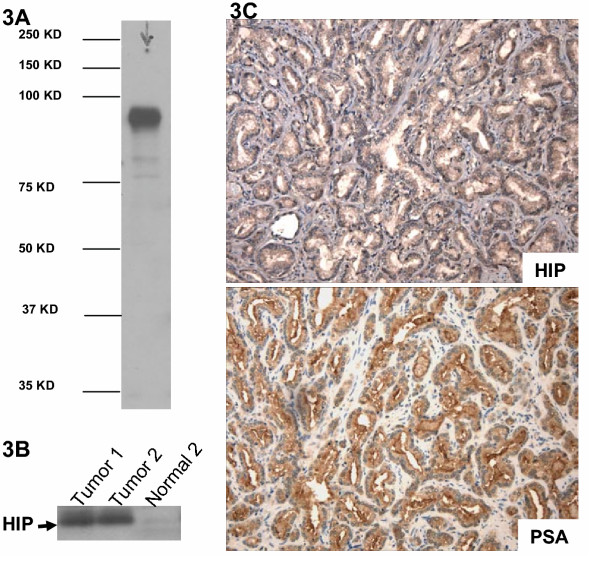
**Detection of HIP in human cancer specimens. **By Western blotting, HIP antibodies (R&D systems Cat# AF1568) recognized one band between 75 and100 KD (**A**). Expression of endogenous HIP was detected in two GI cancer tissues, which were known to contain activated hedgehog signaling (data not shown here), but not in the matched normal tissue (**B**). Immunohistostaining of HIP I prostate cancer showed a similar pattern to PSA (**C**, 200×)

To further confirm our data, we detected HIP protein expression, another marker of the hedgehog signaling activation. After transfection of HIP expressing plasmid into 293 cells, HIP antibodies recognize a single band around 75 KD (Fig. 3A), and an endogenous HIP protein with a similar size was also detected in two cancer tissues, in which hedgehog signaling is known to be activated (Fig. 3B and data not shown here). In contrast, the matched normal tissue did not express detectable HIP. Thus, HIP expression appears to be a good marker for hedgehog signaling activation. Immunohistostaining with HIP antibodies in prostate cancer specimens revealed a similar pattern to prostate specific antigen (PSA) and PTCH1 (Fig. 3C and Table 1, Additional file 1), further confirming that hedgehog pathway is activated in advanced prostate cancers. Thus the hedgehog pathway appears to be frequently activated in advanced or metastatic prostate cancers.

### Altered expression of Su(Fu) and Shh in prostate cancer specimens

**Figure 4:**
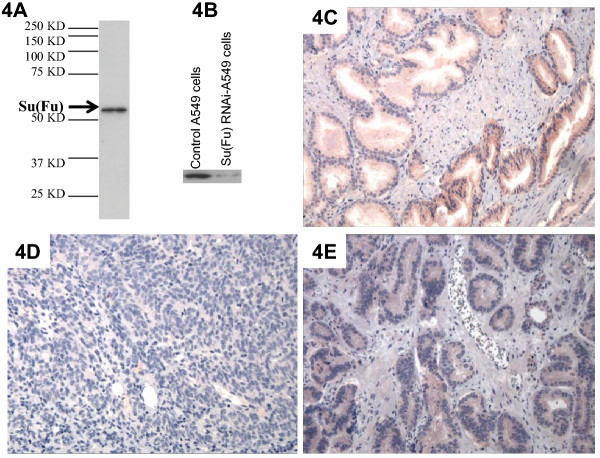
**Detection of Su(Fu) in prostate cancer specimens. **Su(Fu) antibodies (Santa Cruz Biotechnology Cat# 10933) recognized only one single band (54-Kd) in D283 cells (**A**). Following treatment of a specific SiRNA of Su(Fu), the endogenous Su(Fu) band was greatly reduced (**B**). Immunohistostaining with Su(Fu) antibodies in prostate cancer specimens revealed positive (**C**, in red, 200×), negative (**D**, 200×) or weak staining (**E**, red, 200×).

There are several mechanisms by which the hedgehog pathway in these prostate tumors can be activated, including loss of Su(Fu) or over-expression of hedgehog [6-10]. The Su(Fu) gene is localized at 10q24, a region with a frequent LOH in prostate cancer [15,16,18]. Mutations of Su(Fu) have been reported in other human cancers [6]. To test whether loss of Su(Fu) function is responsible for hedgehog signaling activation, we examined expression of Su(Fu) protein in these prostate cancer specimens. The antibodies of Su(Fu) recognize a single band at 52-kD in Western blotting analyses (Fig. 4A), which was reduced following treatment with Su(Fu) SiRNA (Fig. 4B), indicating the specificity of the antibodies. Furthermore, addition of the peptide, from which the antibodies were raised, prevented the antibody binding, further confirming the specificity of our Su(Fu) antibodies (data not shown). Of the 16 PTCH1 positive prostate cancer specimens with Gleason scores 8–10, 9 have no detectable Su(Fu) protein (Fig. 4C,4D,4E and Table 1, Additional file 1). In total, 11 of 27 PTCH1 positive prostate cancer specimens have no detectable Su(Fu) protein. Prostate cancers with low Gleason scores, however, frequently have detectable Su(Fu) protein (see Table 1, Additional file 1), suggesting that loss of Su(Fu) protein may be associated with prostate cancer progression.

**Figure 5:**
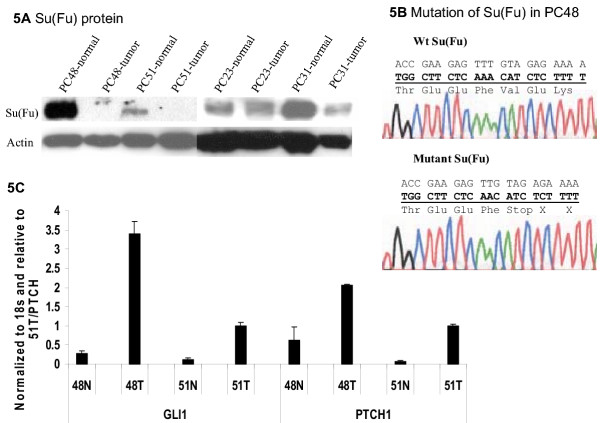
**Inactivation of Su(Fu) in prostate cancer. **Two TURP (Transurethral resection of the prostate) tumors with loss of Su(Fu) expression were confirmed by Western blotting (**A**). One mutation of Su(Fu) found in prostate cancer PC48 is shown in **B**, which is predicted to create a STOP codon in the Su(Fu) coding sequence +1318. The levels of Gli1 and PTCH1 transcripts in prostate tissues were detected by real-time PCR (see methods for details) (**C**). Tumor tissues had higher levels of the target gene transcripts.

To confirm the immunohistochemistry data, we performed immunoblotting analyses using several dissected TURP (Transurethral resection of the prostate) specimens in which tumor portion can be as high as 70% of the tissue mass. Prostatectomy specimens (most of our tumors), however, often contain a small percentage (5–10%) of tumor tissue and are therefore not suitable for Western blotting or real-time PCR analyses. As shown in Fig. 5A, two tumors (PC48 and PC51) had no detectable Su(Fu) protein, which are consistent with our immunohistostaining, suggesting loss of Su(Fu) may be responsible for hedgehog pathway activation in these tumors. The matched normal tissues, however, retained expression of Su(Fu), indicating that alteration of Su(Fu) is a somatic event. Sequence analyses of these two tumors revealed genetic mutations in *Su(Fu)*, which are predicted to create STOP codons in the coding sequence (Fig. 5B and Table 1, Additional file 1). In PC48, a homozygous deletion of A1315 was detected, which results in a STOP codon at +1318 bp (Fig. 5B). In PC51, we detected two types of mutations, one with a deletion of C255, which results in a STOP codon at +294 bp whereas another with a deletion of C198, create a STOP codon (Picture not shown here, see Table 1, Additional file 1). These mutations were confirmed with 6 independent clones from two separate experiments, which exclude the possibility of PCR errors. No mutations were detected from the matched benign tissues, indicating the somatic nature of the mutations. Real-time PCR analyses indicated that target genes of the hedgehog pathway, PTCH1 and Gli1, were all elevated in these tumors (Fig. 5C), confirming activation of the hedgehog pathway in these tumors. Thus, Su(Fu) inactivation appears to contribute to activation of hedgehog signaling in these prostate tumors.

**Figure 2:**
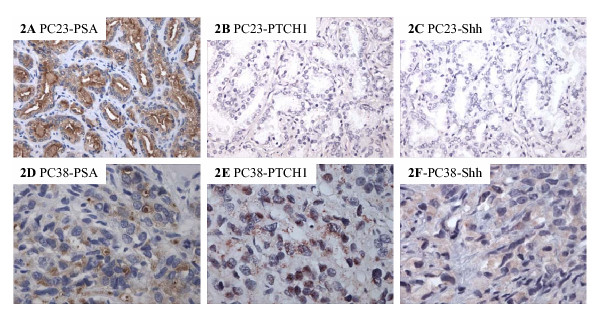
**Co-expression of PTCH1, PSA and Shh in prostate cancer specimens. **Immunohistochemistry of prostate cancer specimens with PSA was used to confirm the cancer region. Positive staining was in red. Positive staining patterns of PTCH1 and Shh antibodies (Santa Cruz Biotechnology Cat# 9024) were similar to that of PSA staining. PC23 (**A-C**) was from tumors with Gleason score 7 (200×). PC38 (**D-F**) was a tumor from Gleason score 10 (400×) (**see **Table 1, Additional file 1 for details).

For tumors with high level of PTCH1 expression, but no changes in Su(Fu) protein expression, we examined expression of sonic hedgehog. It is reported that expression of hedgehog may be responsible for hedgehog signaling activation in lung cancer and GI cancers. Immunohistostaining with sonic hedgehog antibodies indicate that sonic hedgehog is highly expressed in 24 of 27 advanced prostate tumors with elevated expression of PTCH1 and HIP (see Fig. 2 and Table 1, Additional file 1). Thus, activation of the hedgehog pathway, as indicated by elevated PTCH1 and HIP expression, is associated with loss of Su(Fu) expression or elevated hedgehog expression.

### The role for activated hedgehog signaling for cellular functions of prostate cancer

**Figure 6:**
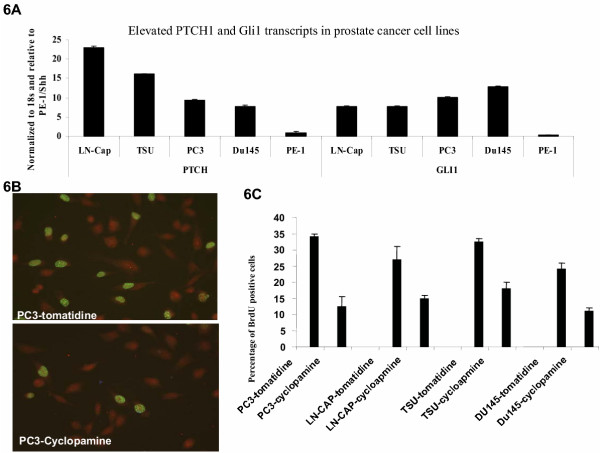
**Cellular functions of the hedgehog pathway in prostate cancer cells**. Expression of hedgehog target genes, PTCH1 and Gli1, were detected by real-time PCR (**A**). DNA synthesis was detected by BrdU labeling (**B**). Over 1000 cells were counted under fluorescent microscope for the percentage of BrdU positive cells, and the experiment was repeated twice (**C**).

To demonstrate the role of hedgehog pathway in prostate cancer, we screen five available cell lines for the expression of Gli1, PTCH1 and HIP. TSU, LNCap, Du145 and PC3 are prostate cancer cell lines whereas RWPE-1 is a prostate epithelial cell line. We found that the hedgehog target genes were significantly elevated in all cancer cell lines (Fig. 6A). Thus, we predicted that inhibition of the hedgehog pathway by smoothened antagonist, cyclopamine, would suppress cell proliferation and cell invasiveness.

Following treatment with 5 μM cycloapmine in PC3 cells, expression of hedgehog target genes were dramatically inhibited (data not shown here), which was accompanied with a significant reduction of BrdU positive cells (see Fig. 6B for details). This effect is specific because addition of tomotidine, a non specific compound with a similar structure to cycloapmine, had no effects on either target gene expression or DNA synthesis (indicated by BrdU labeling in Fig. 6B and 6C). The prostate epithelial RWPE-1 cells which have no activated hedgehog signaling, on the other hand, were not sensitive to cyclopamine (data not shown here), indicating that cyclopamine specifically affects cells with elevated hedgehog signaling. LN-CAP, Du145 and TSU cells, like PC3 cells were also sensitive to cyclopamine treatment (Fig. 6C).

**Figure 7:**
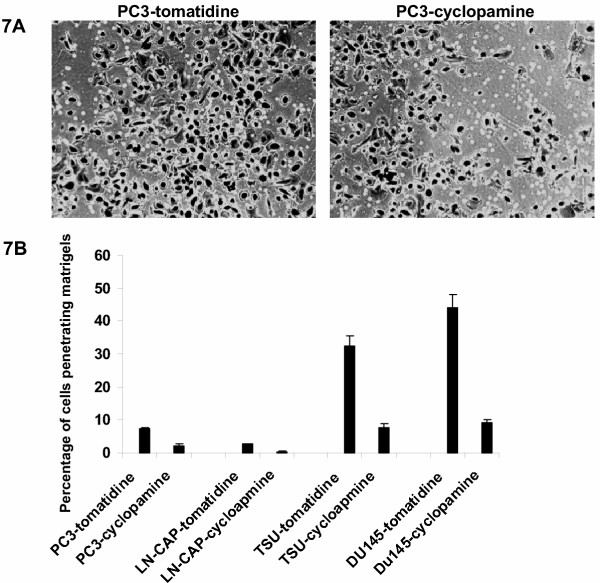
**Effects of cyclopamine on cell invasiveness of prostate cancer cells. **Cell invasion assay of prostate cancer cells was performed using BD Bio-coat cell invasion chambers (**A**). The rate of cell invasion was calculated by dividing cell numbers penetrated the matrigels by the number of cell in the control chambers (without matrigels) (**B**).

Prostate cancer progression is accompanied by increased cell invasiveness. Because the hedgehog signaling activation occurs frequently in advanced prostate cancer, we examined if inhibition of the hedgehog signaling can reduce cell invasiveness. Using BD Bio-coat cell invasion chambers, we found that treatment of cyclopamine in PC3 cells reduced the percentage of invasive cells by 70% (Fig. 7A). Similar data were also observed in Du145, LN-CAP and TSU cells (Fig. 7B). Under the same condition, RWPE-1 cells were not very invasive. Thus, hedgehog signaling activation regulates both cell proliferation as well as cell invasiveness of prostate cancer cells.

**Figure 8:**
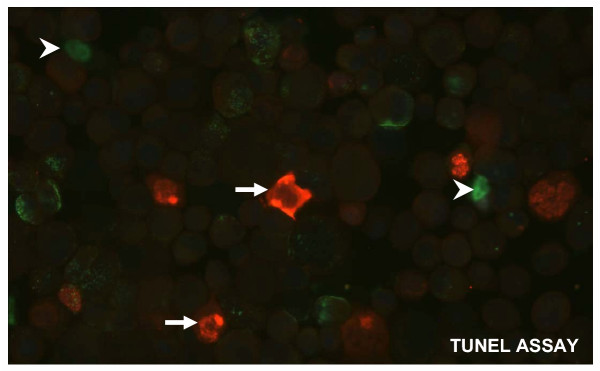
**Cyclopamine induces apoptosis in prostate cancer cells. **Cyclopamine-mediated apoptosis in prostate cancer cells was analyzed by TUNEL assay. TUNEL positive cells were indicated by arrowheads. Cells with expression of Gli1 under the CMV promoter (indicated by the arrows) did not undergo apoptosis (n = 500).

It has been shown that cyclopamine induced apoptosis in cancer cells with activated hedgehog signaling [21]. We have shown that Gli1 down-regulation is necessary for cyclopamine-mediated apoptosis in basal cell carcinoma cells [21]. To test the significant role of Gli1, the down-stream effector and the target gene of the hedgehog pathway, in cyclopamine-mediated apoptosis, we first transfected Gli1 expressing plasmid in to PC3 cells, and then treated the cells with 5 μM cyclopamine for 36 h. Since Gli1 is expressed under the control of the CMV promoter, we predicted that ectopic Gli1-expressing cells should be resistant to apoptosis, which is detected by TUNEL staining. As shown in Fig. 8, we found that all Gli1 positive cells (n = 500) were TUNEL negative, supporting our hypothesis that down-regulation of Gli1 may be an important mechanism by which cyclopamine mediates apoptosis in prostate cancer cells with activated hedgehog signaling.

All these data indicate that the hedgehog pathway is activated in advanced prostate cancers, as indicated by high expression of PTCH1 and HIP. Our results also indicate that hedgehog signaling is required for cell proliferation and cell invasion of prostate cancer cells. Thus, targeted inhibition of the hedgehog pathway may be effective in future prostate cancer therapeutics.

## Discussion

Hedgehog signaling pathway regulates cell proliferation, tissue polarity and cell differentiation during normal development. Abnormal signaling of this pathway has been reported in a variety of human cancers, including basal cell carcinomas, medulloblastomas, small cell lung cancer and GI cancers [3,4,6-10,22,23]. Our findings in this report indicate a role of the sonic hedgehog pathway in prostate cancer. We detected a high expression of hedgehog target genes, PTCH1 and HIP, in advanced or metastatic prostate cancers. In contrast, only 22% of prostate tumors with Gleason scores 3–6 have elevated expression of PTCH1 and HIP. While our manuscript is being reviewed, three independent groups have recently reported similar results [24-26]. Thus, the hedgehog signaling pathway is frequently activated in advanced or metastatic prostate cancers.

### Alterations of genes in the hedgehog pathway in prostate cancer

In our studies, we found that some prostate tumors had no detectable Su(Fu) protein expression while others contained high levels of Shh protein expression. We further identified inactivated mutations of *Su(Fu) *in two prostate cancers. In addition to inactivated mutations in the coding region, Su(Fu) may be inactivated through promoter methylation. The heterogeneous nature of prostate cancer makes it difficult to screen prostate cancer specimens for Su(Fu) mutations since the tumor content is often less than 5% of the specimens. Future improvement can be achieved using microdissection techniques for collecting pure population of tumor cells in gene mutation analysis.

Since all available prostate cancer cell lines express Su(Fu) at a high level, the role of Su(Fu) on cellular functions of prostate cancer cannot be investigated in these cells. It appears that over-expression of sonic hedgehog may be responsible for hedgehog signaling activation in these cell lines [our unpublished data and [24-26]]. After screening over 30 human cancer cell lines, we identified non-prostate cancer cell line with elevated hedgehog target genes and no detectable Su(Fu) expression (data not shown here). The growth suppression effects of Su(Fu) was demonstrated in this cell line, in which Su(Fu) expression down-regulated hedgehog target genes, inhibited DNA synthesis and cell growth (data not shown here). Thus, inactivation of Su(Fu) can contribute to active hedgehog signaling in prostate cancer.

Su(Fu) is reported to affect β-catenin signaling [27,28]. We analyzed expression of β-catenin and E-cadherin in our prostate cancer array and detected cytoplasmic distribution of E-cadherin and β-catenin only in PC51 (data not shown), indicating that Su(Fu) may be able to affect both the wnt pathway and the hedgehog pathway in prostate cancer.

In addition to Su(Fu) inactivation, over-expression of Shh is another mechanism by which the hedgehog pathway is activated in cancer [7-10]. We noticed that sonic hedgehog expression varies from tumor to tumor, which may be resulted from the heterogeneity of prostate cancer. Our immunohistostaining also revealed that three tumors (PC14, PC20 and PC36) expressed PTCH1 and HIP at high levels, but had no alterations of Shh and Su(Fu). This could be due to elevated expression of indian hedgehog, or even alterations of other components of the pathway (such as Rab23 or Fused).

Once hedgehog pathway is activated, the target gene expression will be up-regulated. Thus, analysis of target gene expression using immunohistochemistry will be an effective way to detect hedgehog pathway activation in prostate cancer. Currently, PTCH1, Gli1 and HIP are good markers for the hedgehog pathway.

### Perspectives on prostate cancer therapy

Our findings not only provide novel basic understanding of prostate cancer, but also allow us to design new ways to treat prostate cancer. With a specific SMO antagonist, cyclopamine, it will be possible in the future to treat prostate cancers, which have over-expressed sonic hedgehog. However, as a downstream molecule, tumors with Su(Fu) inactivation may not respond to cyclopamine treatment. Therefore, additional small molecule inhibitors appear to be necessary to treat Su(Fu) inactivated prostate cancer. One possibility is to use Gli1 SiRNA since we have indicated that down-regulation of Gli1 may be an important mechanism by which inhibition of the hedgehog pathway by cyclopamine induces apoptosis (Fig. 8). Sanchez et al also indicated that Gli1 SiRNA down-regulated DNA synthesis in prostate cancer cells [24].

## Conclusion

Taken together, our findings suggest that activation of the hedgehog pathway involves prostate cancer progression. There might be several mechanisms by which the hedgehog pathway is activated in advanced prostate cancers, including loss of Su(Fu) protein expression, over-expression of sonic hedgehog or other alterations. We demonstrate that activation of the hedgehog pathway is associated with DNA synthesis and cell invasiveness in prostate cancer cells. Inhibition of the hedgehog pathway, on the other hand, causes apoptosis possibly through down-regulation of Gli1. Our studies predict that targeted inhibition of the hedgehog pathway may be an effective way to prevent prostate cancer progression.

## Materials and methods

### Tissue Microarray of Prostate Cancer

A total of 55 paraffin-embedded tissue blocks from patients with prostate cancer were obtained from UTMB Surgical pathology with approval from the Institutional Review Board (IRB). Pathological reports, H#E staining of each specimen were reviewed to determine the nature of the disease and the Gleason scores. Of 55 specimens, 18 were from tumors with Gleason scores 3–6, 15 with Gleason score 7 and 22 with Gleason scores 8–10. The tumor area was first identified before tissue microarray (1.5 mm in diameter for specimens) was assembled with Beecher's Tissue arrayer-I^® ^according to manufacturer's instruction .

### Immunohistochemistry and Western blotting

A standard avidin-biotin immunostaining technique was performed using a kit from Vector laboratories using specific antibodies to Su(Fu) (Santa Cruz Biotechnology Cat# 10933), PTCH1 (Santa Cruz Biotechnology Cat# 6149), HIP (R&D systems Cat# AF1568) and Shh (Santa Cruz Biotechnology Cat# 9024) and PSA (Vector laboratories). Positive staining was in red or brown. The specificity of antibodies was tested using the very peptide used for raising the antibodies, which abolished the specific staining. Hematoxylin was used for counterstaining (in blue). Protein was analyzed by Western analysis with appropriate antibodies [Su(Fu) antibodies were from Santa Cruz, beta-actin antibody was purchased from Sigma). The signals were visualized with the enhanced chemiluminescence detection system (Amersham).

### Cell lines and Cell invasion assay

Cell lines (RWPE-1, Du145, PC3, LN-CAP were purchase from ATCC and cultured according to the suggested conditions. TSU was kindly provided by Dr. Allen Gao. Cell invasion assay was performed with BD Bio-coat cell invasion chambers according to manufacturer's instruction (BD Bioscience, Inc., Franklin Lakes, NJ), with triplicates for each sample and the experiment was repeated three times with the similar results. Cell were treated with 5 μM cyclopamine (or tomatidine) before (for 12 h) and during cell invasion assay (for 24 h). The rate of cell invasion was calculated by dividing cell numbers penetrated the matrigels by the number of cell in the control chambers (without matrigels).

### RT-PCR and sequencing analysis

Total RNA was isolated using Trizol^® ^reagent (Invitrogen), and RT-PCR was performed using Promega's RT-PCR system according to the manufacturer's protocol. Two pairs of Su(Fu) primers were used (the first set with the forward primer 5'-cctacgcaccccgatggcg-3" and the reverse primer 5'-agccaaaaccactacctcca-3'; the second set with the forward primer 5'-tccaggttaccgctatcgtc-3' ad the reverse primer 5'-tagtgtagcggactgtcg-3'). PCR products were first purified using Qiagen's Gel Extraction Kit. Due to existence of possible Su(Fu) splicing isoforms in humans, Su(Fu) genetic mutations were screened after the PCR products were cloned into TOPO^® ^TA cloning vectors (Invitrogen). Several independent clones (from three experiments) of each PCR product were selected for sequencing analysis in UTMB sequencing facility. All mutations were confirmed by at least six independent clones.

Real-time PCR We used Applied Biosystems' assays-by-demand 20× assay mix of primers and TaqMan probes (FAM™ dye-labeled) for the target genes (human Gli and PTCH1, the sequences have been patented by Applied Biosystems, Foster City, CA) and pre-developed 18S rRNA (VIC™-dye labled probe) TaqMan^® ^assay reagent (P/N 4319413E) for an internal control. The primers are designed to span exon-exon junctions so as not to detect genomic DNA and the primers and probe sequences were searched against the Celera database to confirm specificity. To obtain the relative quantitation of gene expression, a validation experiment was performed to test the efficiency of the target amplification and the efficiency of the reference amplification. All absolute values of the slope of log input amount vs. ΔC_T _were <0.1. Separate tubes (singleplex) one-step RT-PCR was performed with 20 ng RNA for both target genes and endogenous control. The reagent we used was TaqMan one-step RT-PCR master mix reagent kit (P/N 4309169). The cycling parameters for one-step RT-PCR was: reverse transcription 48°C for 30 min, AmpliTaq activation 95°C for 10 min, denaturation 95°C for 15 sec and annealing/extension 60°C for 1 min (repeat 40 times) on ABI7000. Triplicate C_T _values were analyzed in Microsoft Excel using the comparative C_T_(ΔΔC_T_) method as described by the manufacturer(Applied Biosystems, Foster City, CA). The amount of target (2^-ΔΔCT^) was obtained by normalization to an endogenous reference (18sRNA) and relative to a calibrator.

### BrdU labeling and TUNEL assay

BrdU labeling was performed using an *in situ *cell proliferation kit (Roche Molecular Biochemicals) [22]. Cells were treated with 5μM cyclopamine (or tomatidine) for 12 h before BrdU labeling (1 h at 37°C). The percentage of BrdU positive cells was obtained by counting over 1000 cells under microscope, and the experiment was repeated twice with similar results. TUNEL assay was performed using an *in situ *cell death kit (Roche Molecular Biochemicals) [21,29]. Cells were treated with 5 μM cyclopamine (or tomatidine) for 36 h before TUNEL assay).

## List of abbreviations

PSA – prostate specific antigen; HIP – hedgehog-interacting protein; Su(Fu) – suppressor of fused; PTCH1 – human homologue of *patched *1; Shh – sonic hedgehog; SMO – smoothened, BCC – basal cell carcinoma.

## Authors' contributions

Tao Sheng contributed to Figures 6, 7, 8, cellular functions of the hedgehog pathway in prostate cancer cells. Chegxin Li contributed to primary tumor protein expression, particularly on Su(Fu) expression. Xiaoli Zhang contributed to mutation analyses of Su(Fu) in prostate cancer and real-time PCR analyses. Sumin Chi contributed to HIP antibody test (Fig. 3A and 3B). Nonggao He contributed to HIP antibody staining (Fig. 3C). Kai Chen contributed to PTCH1 antibody test (Fig. 1A). Frank McCormick involved in the initial project discussion. Zoran Gatalica contributed to prostate cancer histology and Gleason scores of the tumors.

## Supplementary Material

Additional File 1 **Table 1 Prostate cancer specimens and protein expression. **Prostate cancer specimens and expression of several hedgehog signaling proteins are summarized in this table (**A**). A total of 55 specimens were used in this study. The Gleason scores and protein expression of Shh, PTCH1 and Su(Fu) are shown (**B**). Click here for file

## References

[B1] Ingham PW (1998). Transducing Hedgehog: the story so far. Embo J.

[B2] Taipale J, Beachy PA (2001). The Hedgehog and Wnt signalling pathways in cancer. Nature.

[B3] Johnson RL, Rothman AL, Xie J, Goodrich LV, Bare JW, Bonifas JM, Quinn AG, Myers RM, Cox DR, Epstein E. H., Jr., Scott MP (1996). Human homolog of patched, a candidate gene for the basal cell nevus syndrome. Science.

[B4] Hahn H, Wicking C, Zaphiropoulous PG, Gailani MR, Shanley S, Chidambaram A, Vorechovsky I, Holmberg E, Unden AB, Gillies S, Negus K, Smyth I, Pressman C, Leffell DJ, Gerrard B, Goldstein AM, Dean M, Toftgard R, Chenevix-Trench G, Wainwright B, Bale AE (1996). Mutations of the human homolog of Drosophila patched in the nevoid basal cell carcinoma syndrome. Cell.

[B5] Xie J, Murone M, Luoh SM, Ryan A, Gu Q, Zhang C, Bonifas JM, Lam CW, Hynes M, Goddard A, Rosenthal A, Epstein E. H., Jr., de Sauvage FJ (1998). Activating Smoothened mutations in sporadic basal-cell carcinoma. Nature.

[B6] Taylor MD, Liu L, Raffel C, Hui CC, Mainprize TG, Zhang X, Agatep R, Chiappa S, Gao L, Lowrance A, Hao A, Goldstein AM, Stavrou T, Scherer SW, Dura WT, Wainwright B, Squire JA, Rutka JT, Hogg D (2002). Mutations in SUFU predispose to medulloblastoma. Nat Genet.

[B7] Berman DM, Karhadkar SS, Hallahan AR, Pritchard JI, Eberhart CG, Watkins DN, Chen JK, Cooper MK, Taipale J, Olson JM, Beachy PA (2002). Medulloblastoma growth inhibition by hedgehog pathway blockade. Science.

[B8] Watkins DN, Berman DM, Burkholder SG, Wang B, Beachy PA, Baylin SB (2003). Hedgehog signalling within airway epithelial progenitors and in small- cell lung cancer. Nature.

[B9] Berman DM, Karhadkar SS, Maitra A, Montes De Oca R, Gerstenblith MR, Briggs K, Parker AR, Shimada Y, Eshleman JR, Watkins DN, Beachy PA (2003). Widespread requirement for Hedgehog ligand stimulation in growth of digestive tract tumours. Nature.

[B10] Thayer SP, Di Magliano MP, Heiser PW, Nielsen CM, Roberts DJ, Lauwers GY, Qi YP, Gysin S, Fernandez-Del Castillo C, Yajnik V, Antoniu B, McMahon M, Warshaw AL, Hebrok M (2003). Hedgehog is an early and late mediator of pancreatic cancer tumorigenesis. Nature.

[B11] Berman DM, Desai N, Wang X, Karhadkar SS, Reynon M, Abate-Shen C, et al. (2004). Roles for Hedgehog signaling in androgen production and prostate ductal morphogenesis.. Dev Biol.

[B12] Podlasek CA, Barnett DH, Clemens JQ, Bak PM, Bushman W (1999). Prostate development requires Sonic hedgehog expressed by the urogenital sinus epithelium. Dev Biol.

[B13] Wang BE, Shou J, Ross S, Koeppen H, De Sauvage FJ, Gao WQ (2003). Inhibition of epithelial ductal branching in the prostate by sonic hedgehog is indirectly mediated by stromal cells. J Biol Chem.

[B14] Freestone SH, Marker P, Grace OC, Tomlinson DC, Cunha GR, Harnden P and Thomson AA. (2003). Sonic hedgehog regulates prostatic growth and epithelial differentiation.. Dev Biol.

[B15] Latini JM, Rieger-Christ KM, Wang DS, Silverman ML, Libertino JA, Summerhayes IC (2001). Loss of heterozygosity and microsatellite instability at chromosomal sites 1Q and 10Q in morphologically distinct regions of late stage prostate lesions. J Urol.

[B16] Leube B, Drechsler M, Muhlmann K, Schafer R, Schulz WA, Santourlidis S, Anastasiadis A, Ackermann R, Visakorpi T, Muller W, Royer-Pokora B (2002). Refined mapping of allele loss at chromosome 10q23-26 in prostate cancer. Prostate.

[B17] Ding Q, Fukami S, Meng X, Nishizaki Y, Zhang X, Sasaki H, et al. (1999). Mouse suppressor of fused is a negative regulator of sonic hedgehog signaling and alters the subcellular distribution of Gli1.. Curr Biol.

[B18] Kogerman P, Grimm T, Kogerman L, Krause D, Unden AB, Sandstedt B, et al. (1999). Mammalian suppressor-of-fused modulates nuclear-cytoplasmic shuttling of Gli-1.. Nat Cell Biol.

[B19] Stone DM, Murone M, Luoh S, Ye W, Armanini MP, Gurney A, Phillips H, Brush J, Goddard A, de Sauvage FJ, Rosenthal A (1999). Characterization of the human suppressor of fused, a negative regulator of the zinc-finger transcription factor Gli. J Cell Sci.

[B20] Meng X, Poon R, Zhang X, Cheah A, Ding Q, Hui CC, Alman B (2001). Suppressor of fused negatively regulates beta-catenin signaling. J Biol Chem.

[B21] Athar M, Li CX, Chi S, Tang X, Zhang X, Kim AL, Tyring SK, Kopelovich L, Epstein EH Jr, Blickers DR, Xie J (2004). Inhibition of smoothened signaling prevents ultraviolet B-induced basal cell carcinomas through induction of fas expression and apoptosis.. Cancer Res.

[B22] Xie J, Aszterbaum M, Zhang X, Bonifas JM, Zachary C, Epstein E, McCormick F (2001). A role of PDGFRalpha in basal cell carcinoma proliferation. Proc Natl Acad Sci U S A.

[B23] Xie J, Johnson RL, Zhang X, Bare JW, Waldman FM, Cogen PH, Menon AG, Warren RS, Chen LC, Scott MP, Epstein E. H., Jr. (1997). Mutations of the PATCHED gene in several types of sporadic extracutaneous tumors. Cancer Res.

[B24] Sanchez P, Hernandez AM, Stecca B, Kahler AJ, DeGueme AM, Barrett A, et al. (2004). Inhibition of prostate cancer proliferation by interference with SONIC HEDGEHOG-GLI1 signaling.. Proc Natl Acad Sci U S A.

[B25] Karhadkar SS, Steven Bova G, Abdallah N, Dhara S, Gardner D, Maitra A, et al. (2004). Hedgehog signalling in prostate regeneration, neoplasia and metastasis.. Nature.

[B26] Fan L, Pepicelli CV, Dibble CC, Catbagan W, Zarycki JL, Laciak R, et al. (2004). Hedgehog signaling promotes prostate xenograft tumor growth.. Endocrinology.

[B27] Meng X, Poon R, Zhang X, Cheah A, Ding Q, Hui CC and Alman B. (2001). Suppressor of fused negatively regulates beta-catenin signaling.. J Biol Chem.

[B28] Taylor MD, Zhang X, Liu L, Hui CC, Mainprize TG, Scherer SW, et al. (2004). Failure of a medulloblastoma-derived mutant of SUFU to suppress WNT signaling.. Oncogene.

[B29] Li C, Chi S, He N, Zhang X, Guicherit O, Wagner R, Tyring S, Xie J (2004). IFNalpha induces Fas expression and apoptosis in hedgehog pathway activated BCC cells through inhibiting Ras-Erk signaling. Oncogene.

