# Supplementary material for: Activation of the hedgehog pathway in advanced prostate cancer
Source: Mol Cancer. 2004 Oct 13;3:29. doi: 10.1186/1476-4598-3-29 (PMC524523; doi:10.1186/1476-4598-3-29)
Supplement: Additional File 1 — Table 1 Prostate cancer specimens and protein expression. Prostate cancer specimens and expression of several hedgehog signaling proteins are summarized in this table (A). A total of 55 specimens were used in this study. The Gleason scores and protein expression of Shh, PTCH1 and Su(Fu) are shown (B). [file 1476-4598-3-29-S1.doc]

### Table 1 Prostate cancer specimens and protein expression

1. **Summary**

| Tumor grade | | | | | | | | | | | |
| --- | --- | --- | --- | --- | --- | --- | --- | --- | --- | --- | --- |
| Gleason 3-6 (**18**) | | | | Gleason 7 (**15**) | | | | Gleason 8-10 (**22**) | | | |
| 18  Su(Fu)  Positive | 4  PTCH1  Positive | 4  HIP positive | 10  Shh positive | 13  Su(Fu)  Positive | 7  PTCH1  Positive | 7  HIP positive | 4  Shh positive | 13  Su(Fu)  Positive | 16  PTCH1  Positive | 16  HIP positive | 14  Shh positive |

B. Cancer specimens and protein expression

| Specimens | Gleason Score | Su(Fu) protein | PTCH1 protein | HIP protein | Shh Protein |
| --- | --- | --- | --- | --- | --- |
| PC1 | G3 | + | _ | _ | + |
| PC2 | G4 | + | _ | _ | + |
| PC3 | G4 | + | _ | _ | _ |
| PC4 | G4 | + | _ | _ | + |
| PC5 | G5 | + | _ | _ | _ |
| PC6 | G5 | + | + | + | + |
| PC7 | G5 | + | _ | _ | + |
| PC8 | G5 | + | _ | _ | + |
| PC9 | G5 | + | _ | _ | _ |
| PC10 | G6 | + | _ | _ | _ |
| PC11 | G6 | + | + | + | + |
| PC12 | G6 | + | _ | _ | _ |
| PC13 | G6 | + | _ | _ | _ |
| PC14 | G6 | + | + | + | _ |
| PC15 | G6 | + | _ | _ | + |
| PC16 | G6 | + | _ | _ | _ |
| PC17 | G6 | + | _ | _ | + |
| PC18 | G6 | + | + | + | + |
| PC19 | G7 | + | + | + | + |
| PC20 | G7 | + | + | + | _ |
| PC21 | G7 | + | _ | _ | _ |
| PC22 | G7 | _ | + | + | _ |
| PC23 | G7 | + | _ | _ | _ |
| PC24 | G7 | + | _ | _ | _ |
| PC25 | G7 | + | _ | _ | _ |
| PC26 | G7 | + | _ | _ | _ |
| PC27 | G7 | + | _ | _ | _ |
| PC28 | G7 | _ | + | + | _ |
| PC29 | G7 | + | _ | _ | _ |
| PC30 | G7 | + | + | + | + |
| PC31 | G7 | + | _ | _ | _ |
| PC32 | G7 | + | + | + | + |
| PC33 | G7 | + | + | + | + |
| PC34 | G8 | + | _ | _ | _ |
| PC35 | G8 | + | _ | _ | + |
| PC36 | G8 | + | + | + | _ |
| PC37 | G8 | _ | + | + | + |
| PC38 | G8 | + | + | + | + |
| PC39 | G9 | + | + | + | + |
| PC40 | G9 | + | + | + | + |
| PC41 | G9 | _ | + | + | + |
| PC42 | G9 | + | + | + | + |
| PC43 | G9 | + | _ | _ | _ |
| PC44 | G9 | + | _ | _ | _ |
| PC45 | G9 | + | _ | _ | _ |
| PC46 | G9 | _ | + | + | _ |
| PC47 | G9 | _ | + | + | _ |
| PC48# | G9 | _ | + | + | + |
| PC49 | G9 | + | + | + | + |
| PC50 | G10 | + | _ | _ | _ |
| PC51# | G10 | _ | + | + | + |
| PC52 | G10 | _ | + | + | + |
| PC53 | G10 | _ | + | + | + |
| PC54 | G10 | + | + | + | + |
| PC55 | G10 | _ | + | + | + |
| PC56 | Metastatic | + | + | + | + |
| PC57 | Metastatic | _ | + | + | + |
| PC58 | Metastatic | _ | + | + | + |
| PC59 | Metastatic | + | + | + | + |

# Inactivated mutations of Su(Fu) were detected in PC48 and PC51. PC48 contains a homogeneous A1315 deletion, resulting in a STOP codon at +1318. PC51 contains two point mutations, one with a deletion of C255, which results in a STOP codon at +294bp and another with a deletion of C198, creating a STOP codon.
